# *Bartonella*, *Rickettsia*, *Babesia*, and *Hepatozoon* Species in Fleas (Siphonaptera) Infesting Small Mammals of Slovakia (Central Europe)

**DOI:** 10.3390/pathogens11080886

**Published:** 2022-08-06

**Authors:** Eva Špitalská, Lenka Minichová, Zuzana Hamšíková, Michal Stanko, Mária Kazimírová

**Affiliations:** 1 Institute of Virology, Biomedical Research Center, Slovak Academy of Sciences, Dúbravská cesta 9, 845 05 Bratislava, Slovakia; 2 Institute of Zoology, Slovak Academy of Sciences, Dúbravská cesta 9, 845 06 Bratislava, Slovakia; 3 Institute of Parasitology, Slovak Academy of Sciences, Hlinkova 3, 040 01 Košice, Slovakia

**Keywords:** *Bartonella*, *Rickettsia*, *Babesia*, *Hepatozoon*, fleas, small rodents, Slovakia

## Abstract

Fleas (Siphonaptera) as obligate, blood-feeding ectoparasites are, together with ticks, hosted by small mammals and can transmit causative agents of serious infections. This study aimed to determine and characterize the presence and genetic diversity of *Bartonella*, *Rickettsia*, and apicomplexan parasites (*Babesia*, *Hepatozoon*) in fleas feeding on small mammals from three different habitat types (suburban, natural, and rural) in Slovakia. The most common pathogen in the examined fleas was *Bartonella* spp. (33.98%; 95% CI: 30.38–37.58), followed by *Rickettsia* spp. (19.1%; 95% CI: 16.25–22.24) and apicomplexan parasites (4.36%; 95% CI: 2.81–5.91). *Bartonella* strains belonging to *B. taylorii*, *B. grahamii*, *B. elizabethae*, *Bartonella* sp. wbs11, and *B. rochalimae* clades were identified in *Ctenophthalmus agyrtes*, *C. congener*, *C. assimilis*, *C. sciurorum*, *C. solutus*, *C. bisoctodentatus*, *Palaeopsylla similis*, *Megabothris turbidus*, and *Nosopsyllus fasciatus* within all habitats. The presence of *Rickettsia helvetica*, *R. monacensis*, and rickettsiae, belonging to the *R. akari* and *R. felis* clusters, and endosymbionts with a 96–100% identity with the *Rickettsia* endosymbiont of *Nosopsyllus laeviceps laeviceps* were also revealed in *C. agyrtes*, *C. solutus*, *C. assimilis*, *C. congener*, *M. turbidus*, and *N. fasciatus*. *Babesia* and *Hepatozoon* DNA was detected in the fleas from all habitat types. *Hepatozoon* sp. was detected in *C. agyrtes*, *C. assimilis*, and *M. turbidus*, while *Babesia microti* was identified from *C. agyrtes*, *C. congener*, and *P. similis*. The present study demonstrated the presence of zoonotic pathogens in fleas, parasitizing the wild-living small mammals of southwestern and central Slovakia and widens our knowledge of the ecology and genomic diversity of *Bartonella*, *Rickettsia*, *Babesia*, and *Hepatozoon*.

## 1. Introduction

Fleas (Siphonaptera) are obligate, blood-feeding ectoparasites. They are both vectors and hosts to pathogenic microorganisms and parasites of medical and veterinary importance, playing a role in their dispersal. Pathogens and parasites can be transmitted by fleas by the oral route through the regurgitation of blood meals, by the fecal route through contaminated fecal pellets and in some cases, hosts acquire infection by consuming infected fleas [1]. Fleas change hosts and come into contact with domestic animals as well as humans and can potentially infect them. For that reason, it is important to know which zoonotic agents are present in fleas.

The most serious human infection spread by fleas is plague, which is caused by *Yersinia pestis*. Fleas are known vectors of *Rickettsia typhi*, which causes murine typhus, and *Bartonella henselae*, which causes cat scratch disease, and also play a role in the transmission of epidemic typhus caused by *Rickettsia prowazekii.* Flea-borne spotted fever and its agent *Rickettsia felis* occur throughout the world. Additionally, fleas host endoparasitic helminths [1]. The major hosts of fleas are mammals, mainly rodents, shrews, hedgehogs, lagomorphs, bats, carnivores, ungulates, and birds, which are also hosts of ticks. Ticks are known vectors of different zoonotic species of *Rickettsia* and *Babesia* and are carriers of *Bartonella* species. Fleas can potentially ingest tick-borne microorganisms from infected hosts or acquire them through co-feeding with ticks on the same host [2,3]. However, whether fleas can also be involved in the transmission cycles of tick-borne pathogens in natural foci has not been proven.

*Bartonella* species are Gram-negative bacteria and are of medical and veterinary importance. They infect the erythrocytes and endothelial cells of mammals. *Bartonella henselae*, *B. quintana*, and *B. bacilliformis* have been found to be responsible for the majority of human bartonellosis cases, whereby *B. henselae* is the causative agent of the well-known zoonotic infection called cat scratch disease [4]. *Bartonella* are transmitted through the bite or scratch of infected animals or by different ectoparasitic arthropods, including fleas, lice, and sand flies. The vector role of ticks and mites or of biting and ectoparasitic dipterans have been suggested but not definitely proven [5]. The cat flea (*Ctenocephalides felis*) is the main vector for *B. henselae.* Other flea species may also transmit *B. henselae* or other *Bartonella* spp.; however, their vector role needs to be confirmed by vector competence studies [5]. Cats, dogs, and rodents are considered natural reservoirs of *Bartonella* spp. in Europe [6]. In Slovakia, knowledge of the occurrence of human bartonellosis is limited. However, during a recent serosurvey in eastern Slovakia, which included 536 people, positivity against anti-*B. henselae* and anti-*B. quintana* antibodies were detected in 23.5% and 24.8% of the examined humans, respectively [7]. Presence of genotypes belonging to *Bartonella taylorii*, *B. rochalimae*, *B. elizabethae*, *B. grahamii*, *B. birtlesii*, and *Bartonella* sp. wbs11 clades was demonstrated in the spleens of small mammals in Slovakia, including those from which the fleas examined in the present study were collected [8,9].

*Rickettsia* species are obligate intracellular Gram-negative bacteria and are responsible for mild to severe diseases in humans, with a worldwide distribution [10]. They are classically transmitted to humans by arthropod vectors such as ticks, mites, fleas, and lice. The associations of rickettsiae with different groups of ectoparasitic arthropods are diverse. In Slovakia, *Rickettsia helvetica*, *R. monacensis*, *Candidatus* Rickettsia mendelii, and *Candidatus* Rickettsia vini, were identified in host-seeking and host-feeding *Ixodes ricinus* ticks from different habitats [11,12,13,14,15]. The presence of *R. helvetica* and *R. monacensis* was also confirmed in the tissue samples of small rodents (*Apodemus flavicollis*, *Myodes glareolus*, and *Microtus arvalis*) [14,16], and *Rickettsia slovaca* and *Rickettsia raoultii* were detected in *Dermacentor marginatus* and *Dermacentor reticulatus* ticks [14,15,17,18]. In *Haemaphysalis inermis* ticks, *Candidatus* Rickettsia hungarica was identified in Slovakia for the first time in 2018 [15]. In the fleas, mites, and ticks infesting different species of small mammals from eastern Slovakia, *Rickettsia helvetica*, unidentified *Rickettsia* sp., and rickettsial endosymbionts were detected [16,19,20], and *Rickettsia felis* DNA was reported for the first time in a *Ctenophthalmus solutus* flea collected from *Apodemus agrarius* [19]. In addition, the DNA of *Rickettsia* sp., closely related to *R. felis*, was detected in *A. flavicollis* from the same region [16]. The presence of *R. africae*, usually transmitted by ticks, was identified in *Ceratophyllus garei* fleas collected after a blood meal on reed warblers (*Acrocephalus scirpaceus*) that were crossing Slovakia during their spring migration [21].

Babesiosis is a tick-borne zoonotic disease caused by protozoan parasites of the genus *Babesia*. These parasites infect a wide range of domestic and wild animals and are transmitted by ixodid ticks of several genera [22]. In previous studies, *Babesia microti* (strain Jena) and *B. venatorum* were identified in host-seeking *I. ricinus* and *Babesia canis* in *D. reticulatus* ticks in southwestern (SW) Slovakia [18,23]. The same zoonotic strain of *B. microti* was also found in rodents and rodent-attached *I. ricinus* ticks [23]. Although *B. microti* is considered a tick-borne pathogen, its presence has been sporadically detected, e.g., in fleas collected from cats and dogs in Poland [24] or in fleas recovered from wild rodents in the USA [3]. In addition, bank voles (*Myodes glareolus*) in SW Slovakia were found infected with two genetic variants of *Hepatozoon* sp., *Hepatozoon* sp. SK1, and *Hepatozoon* sp. SK2 [25]. These correspond to the genotypes UR1 and UR2 of *Hepatozoon erhardovae*, a common blood parasite of bank voles, which is associated with fleas [26,27].

In the present study, we aimed to examine fleas collected from wild rodents for the presence of *Bartonella*, *Rickettsia*, *Babesia*, and *Hepatozoon* species and determine their potential role in the transmission of etiological agents of zoonotic diseases in three habitat types in Slovakia: Bratislava forest park and a patch of deciduous forest remnants in the campus of the Slovak Academy of Sciences (suburban), a deciduous forest at Fúgelka, located in the Small Carpathian Mountains (natural), and a forest-steppe rural area in the Prievidza district.

## 2. Results

### 2.1. Parasitological Aspects of Infestation of Rodents by Fleas

In total, 734 flea specimens from 12 species were recovered from the captured rodents: 434 in the suburban habitat, 257 in the natural habitat, and 43 in the rural habitat (Table S1). Seven species were identified in the suburban habitat, eight in the natural habitat, and five in the rural habitat (Table S2). The total prevalence and mean intensity of infestation of rodents with fleas were 45.47% and 2.44, respectively. The highest prevalence was found in the suburban habitat (52.33%), followed by the rural habitat (40.35%) and the natural habitat (39.67%). The highest mean intensity of infestation was found in the suburban habitat (2.76) and the lowest in the rural habitat (1.87) (Table S1).

### 2.2. Bartonella, Rickettsia, Babesia, and Hepatozoom Species in Fleas

A total of 665 fleas represented by 12 species were analyzed for the presence of pathogenic microorganisms (Table 1, Table 2 and Table 3). The most common pathogen in the sample set of tested fleas was *Bartonella* spp. (33.98%; 95% CI: 30.38–37.58), followed by *Rickettsia* spp. (19.1%; 95% CI: 16.25–22.24) and *Babesia* and *Hepatozoon* spp. (4.36%; 95% CI: 2.81–5.91). The same order of infection rates was found in the rural habitat: *Bartonella* spp. (80%; 95% CI: 68.31–91.69, *Rickettsia* spp, (57.78%; 95% CI: 43.35–72.21), and *Babesia* and *Hepatozoon* spp. (2.22%; 95% CI: 0–6.53). However, in the natural habitat, a higher proportion of the fleas were infected with *Babesia* and *Hepatozoon* spp. (8.62%; 95% CI: 5.01–12.23) than with *Rickettsia* (0.86%; 95% CI: 0–2.05). In the suburban habitat, the prevalence of *Rickettsia* spp. (25.52%; 95% CI = 21.18–29.85) was higher than that of *Bartonella* spp. (22.94%; 95% CI: 18.75–27.12). There was no significant difference between the infection rates in flea males and females. Details are provided below and summarized in Table 1, Table 2 and Table 3.

#### 2.2.1. Prevalence and Diversity of *Bartonella* Species

*Bartonella* DNA was detected in 226 fleas (97 males, 129 females). The difference in *Bartonella* overall prevalence, between flea males and females, was not statistically significant (*p* = 0.6214). The overall infection rate was 33.98%, ranging from 22.94% in the suburban habitat to 80% (95% CI: 68.31–91.69) in the rural habitat (Table 1). Infection rates differed significantly between all habitats (*p* < 0.001). The difference in prevalence between males and females was not significant in any of the studied habitats (natural: *p* = 0.8952, rural: *p* = 0.4487, suburban: *p* = 0.3261). In the suburban and natural habitats, *C. congener* was the most infected species with *Bartonella*, 35.9% (95% CI: 20.84–50.95) and 48.28% (95% CI: 30.09–66.46), respectively, when not considering species with low numbers (Table 1). In the rural habitat, all examined *Ctenophtalmus assimilis* (8 specimens) and *Megabothris turbidus* (1 specimen) were positive for *Bartonella* DNA. Besides, high infection rates were recorded in *Ctenophtalmus solutus* (81.82%, 95% CI: 65.70–97.94) and in *Nosopsyllus fasciatus* (85.71%, 95% CI: 59.79–111.64).

Sequence analysis of the 16S–23S rRNA intergenic spacer region in 145 randomly selected flea DNA lysates showed that the *Bartonella* strains belonged to multiple clusters. *Bartonella* spp. in the fleas from all three habitats belonged to four clusters: *B. taylorii*, *B. grahamii*, *Bartonella* sp. wbs11, and *B. rochalimae.* In addition, in fleas from the rural habitat, *Bartonella*, belonging to the *B. elizabethae* cluster, were identified. Sequences from this study are identical to those previously identified in rodents from the same study sites [9] and were deposited in GenBank. The majority of the DNA sequences were ascribable to the *B. taylorii* cluster (57 flea specimens from the suburban habitat: 36 *Ctenophtalmus agyrtes*, 13 *Ctenophtalmus congener*, 2 *Ceratophylus sciurorum*, 6 *Ctenophtalmus solutus*; 45 specimens from the natural habitat: 9 *C. congener* 34 *C. agyrtes*, 1 *Ctenophtalmus bisoctodentatus*, 1 *Paleopsylla similis*; and 10 specimens from the rural habitat: 5 *C. solutus*, 4 *C. assimilis*, 1 *M. turbidus*). The number of base substitutions in the sequences, per habitat, are shown in Supplementary Materials File S1.

Thirteen sequences (obtained from 3 *C. solutus* specimens from the suburban habitat, 3 *C. agyrtes* from the natural habitat, and 2 *C. assimilis*, 4 *C. solutus*, and 1 *C. agyrtes* from the rural habitat) belonged to the cluster of uncultured *Bartonella* sp. (DQ155376) and uncultured *Bartonella* sp. clone PD134, PD135, PD139 (MF039574- MF039576) identified in the rodents from the rural habitat [9], with a 98–100% identity and similar to those of *B. rochalimae* (95% identity; KU292577) and *B. clarridgeiae* (91–92% identity; AF312497). The sequence from a *C. solutus* specimen from the suburban habitat (MK239961) was 85% identical to the uncultured *Bartonella* sp. clone PD174 (MF039578) [9] and 76% identical to *B. rochalimae* (KU292577) (see phylogenetic tree in Figure S1).

The next cluster was created by eight sequences derived from one *C. agyrtes* from the rural habitat, one *C. agyrtes* from the suburban habitat, and six *C. agyrtes* specimens from the natural habitat, with a 97–100% identity with uncultured *Bartonella* sp. wbs11, a rodent-associated species (AJ269792) identified in the blood of *Apodemus sylvaticus* from the United Kingdom [28], and an uncultured *Bartonella* sp. clone PD130 (MF039573) and clone FU241 (MF039566), originating from rodent samples from the natural and rural habitats of Slovakia [9]. Two sequences (derived from 1 *C. agyrtes* from the natural habitat and 1 *C. sciurorum* from the suburban habitat) were 95–98% identical with *B. doshiae* (AJ269786). In the next cluster, five sequences (derived from 1 *M. turbidus* and 1 *C. agyrtes* from the natural habitat, 1 *M. turbidus* from the suburban habitat, and 1 *N. fasciatus* from the rural habitat) were 98–100% identical to *B. grahamii* (AJ269785), which was previously identified in the blood of woodland rodents in the United Kingdom [28] and to the uncultured *Bartonella* sp. clone FU80 (MF039560) isolated from the spleen of *Apodemus flavicollis* and *Myodes glareolus* found in the natural habitat [9]. In one *C. agyrtes* specimen from the natural habitat, the sequence (MK239960) was 91% identical to *B. grahamii* and uncultured *Bartonella* sp. FU80.

Four sequences derived from three *N. fasciatus* specimens and one *C. assimilis* specimen from the rural habitat were 99–100% identical to *B. elizabethae* (JF766264) isolated from *Meriones libycus* heart tissue in Georgia [29] and to uncultured *Bartonella* sp. clone PD173 (MF039577) isolated from the spleen of *A. flavicollis* trapped in a rural habitat [9].

#### 2.2.2. Prevalence and Diversity of *Rickettsia* Species

The presence of *Rickettsia* DNA was found in 127 fleas (59 males, 68 females), with a total prevalence of 19.1% without a significant difference (*p* = 0.620) between the males and females. The overall infection rate ranged from 0.86% in the natural habitat to 57.78% (95% CI: 43.35–72.21) in the rural habitat and 25.52% in the suburban habitat (Table 2). The differences in prevalence between males and females were not significant in any of the habitats (natural: *p* = 1.0, rural: *p* = 0.766, suburban: *p* = 0.155). The infection rates differed significantly between all habitats (*p* < 0.001). In the suburban and rural habitats, *C. solutus* was the most infected species (32.14%, 95% CI: 19.91–44.38 and 77.27%, 95% CI: 59.76–94.78, respectively), followed by *C. agyrtes* (25.9%, 95% CI: 20.75–31.05) and *N. fasciatus* (57.14, 95% CI: 20.48–93.80), respectively. *C. agyrtes* was the only species infected with rickettsiae in the natural habitat, with a prevalence of 1.03% (95% CI: 0–2.45).

Sequence analysis of the fragment of the *gltA* gene in *Rickettsia*–positive fleas showed that the sequences from eight specimens (7 *C. agyrtes*, 1 *C. solutus*) from the suburban habitat, two *C. agyrtes* specimens from the natural habitat, and three specimens (1 *N. fasciatus*, 1 *C. solutus*, 1 *M. turbidus*) from the rural habitat, were identical with *Rickettsia helvetica* M31 IR SAV (MF673863) and *R. helvetica* (KY488349) (see phylogenetic tree in Figure S2), and were confirmed using *R. helvetica*-specific real-time PCR assay. Fragments of the *gltA* and 17-kDA genes identified in a *N. fasciatus* a *C. assimilis* specimen from the rural habitat belonged to the *Rickettsia felis* cluster, and three *C. solutus* and one *C. assimilis* specimens belonged to the *Rickettsia akari* cluster (*R. akari* str. Hartford CP000847) (see phylogenetic trees in Figures S2 and S3). A fragment of *gltA* gene from a *C. agyrtes* specimen from the rural habitat was also identical to *R. akari* str. Hartford (CP000847); however, amplification of the fragments of the *ompA*, *sca4*, and 17-kDa genes was not successful. A fragment of the *gltA* gene from a *C. agyrtes* specimen from the suburban habitat was identical with *Rickettsia monacensis* isolate 31IRM BA (KF258159), previously identified in a host-seeking *Ixodes ricinus* male collected in Bratislava [12]. Fragments of the *gltA* and 17-kDA genes from a *C. solutus* specimen from the suburban habitat belonged to the cluster of *Rickettsia slovaca*, *R. raoultii*, *R. conori*, *R. sibirica* (MH064462, MG190324, U59728, MF002541 of the *gltA* gene, and KY069263, KX506726, AE006914, MF002549 of the 17-kDA gene) (Figures S2 and S3), but *ompA* and *sca4* genes could not be amplified. Sequence analysis of the fragments of the *gltA* gene in 56 *C. agyrtes*, 12 *C. solutus*, and 3 *C. congener* specimens from the suburban habitat, and 2 *N. fasciatus* and 2 *C. solutus* specimens from the rural habitat, showed a 96–100% identity with *Rickettsia* endosymbionts of *Nosopsyllus laeviceps laeviceps* from China (KX457954), and also with a *Rickettsia* endosymbiont of *Empis bicuspidata* (JQ925616), *Gymnopternus brevicornis* (JQ925600), *Gymnopternus metallicus* (JQ925549), *Argyra vestita* (JQ925587), and *Tetranychus urticae* (KP828066) (Figure S2). Unfortunately, fragments of *ompA*, *sca4*, and 17-kDa genes in these samples were not of a good enough quality for subsequent analyses.

#### 2.2.3. Prevalence and Diversity of *Babesia* and *Hepatozoon* Species

*Babesia* and *Hepatozoon* DNA were detected in 29 fleas (13 males, 16 females), with a total prevalence of 4.36% (Table 3). The difference in prevalence between male and female fleas was not significant (*p* = 1.0). The highest prevalence (8.62%; 95% CI: 5.01–12.23) was documented in fleas from the natural habitat, followed by the rural habitat (2.22%; 95% CI: 0–6.53) and the suburban habitat (2.06%; 95% CI: 0.65–3.48). Prevalence differed significantly only between the natural and suburban habitats (*p* < 0.001). Among the more abundant species, *C. agyrtes* was the most infected in the natural habitat (8.25%; 95% CI: 4.38–12.12), followed by *C. congener* (6.90%; 95% CI: 0–16.12), whereas in the suburban habitat, the infection rates of these two species were similar (*C. agyrtes* 2.52%; 95% CI: 0.68–4.36; *C. congener* 2.56%; 95% CI: 0–7.52). In the rural habitat, *C. assimilis* was the only infected species (12.5%; 95% CI: 0–35.42).

*Babesia microti* and *Hepatozoon* sp. were identified in 15 (51.72%) and 14 (48.23%) positive fleas, respectively. *Babesia microti* was detected in five *C. agyrtes* specimens from the suburban habitat and six *C. agyrtes* specimens from the natural habitat, with one and two *C. congener* specimens from the suburban and the natural habitat found, respectively, and also in one *P. simillis* specimen from the natural habitat. *Hepatozoon* sp. was found in fleas from all studied habitats (rural: 1 *C. assimilis*; suburban: 2 *C. agyrtes*; and natural: 10 *C. agyrtes*, 1 *M. turbidus*). Sequence analysis of the amplified 18S rRNA fragments from the 15 isolates in the fleas revealed a 100% identity with the sequences of *B. microti* (strain Jena) from host-seeking and rodent-attached *I. ricinus* ticks (e.g., KU550676 and KU550680) and rodents (e.g., KU362894, KU362896) originating from the same study sites in the suburban and natural habitats [23]. Sequences from 14 isolates from fleas were 100% identical with *Hepatozoon* sp. SK1, previously identified in *M. glareolus* from the suburban (KU597243) and natural (KU597245) habitats [25].

#### 2.2.4. Coinfections

In all types of habitats, the co-infections of two pathogens were recorded (Table 4). The total co-infection rate was 7.52%, whereby the highest rate was detected for *Bartonella* spp. and rickettsial endosymbionts. The co-infection of three pathogens (*R. helvetica*, *B. microti* and *Bartonella* from cluster *B. taylorii*) was recorded only in a *C. agyrtes* specimen from the suburban habitat.

## 3. Discussion

The investigation of communities of fleas that have infested different mammal and bird species has a long tradition in Slovakia, while studies about zoonotic pathogens transmitted and/or carried by fleas are limited. With regards to the flea species spectrum, prevalence, and intensity of infestation in small rodents, we confirmed the previous findings [30,31,32] and have not detected any new flea–rodent host associations. We identified *Bartonella* strains belonging to *B. taylorii*, *B. grahamii*, *B. elizabethae*, and *Bartonella* sp. wbs11, and *B. rochalimae* clades, *R. helvetica*, *R. monacensis*, and rickettsiae, belonging to *R. akari* and *R. felis* clusters and the cluster of *R. slovaca*, *R. raoultii*, *R. conorii*, and *R. sibirica*, endosymbionts, finding an identity with a *Rickettsia* endosymbiont of *Nosopsyllus laeviceps laeviceps*, *B. microti*, and *Hepatozoon* sp. in fleas feeding on small rodents.

The ecological and bacteriological observations of small mammals and their ectoparasites from different regions and habitat types in Slovakia have shown abundant and diverse communities of microorganisms. In our previous studies, which comprise the same habitats as the present study, we focused mainly on ticks, and analyzed host-seeking *Haemaphysalis concinna*, *I. ricinus*, and *D. reticulatus* ticks and *Ixodes trianguliceps*, *I. ricinus*, *H. concinna*, and *D. reticulatus* ticks, collected from vertebrate hosts as well as blood and tissue samples from small mammals, birds, and free-living ungulates, searching for the presence of rickettsiae, bartonellae, and babesiae [9,12,13,14,33].

Our findings on the diversity of *Bartonella* spp. in fleas corresponds with the species spectrum found in rodent spleen samples [9], which points to the circulation of *Bartonella* spp. between small rodents and fleas in the explored habitats, where all analyzed host-seeking *I. ricinus* ticks were negative [9]. The overall prevalence of *Bartonella* spp. in rodents was 64.8%; the highest rate of 73.8% was found in the natural habitat, and the lowest one (56.0%) in the suburban habitat [9]. In the tested fleas, the overall prevalence was 33.98%; the highest rate of 80% was found in the rural habitat and could probably be related to the low numbers of tested fleas in comparison with the other habitats. *Bartonella taylorii* was the most common species identified in the fleas from this study, similarly to the rodents from Slovakia [8,9] and the fleas associated with small rodents and rodents in other European countries [34,35,36]. This species can cause infection in animals; however, its pathogenic potential in humans is unknown [35] but should be considered. *Bartonella* strains belonging to the *B. grahamii*, *B. elizabethae*, and *B. rochalimae* clades, which were detected in both fleas and rodents from Slovakia, may also represent a potential risk for humans. For example, *B. grahamii* is a causative agent of human renitis [37], and *B. elizabethae* causes endocarditis [38], with *B. rochalimae* causing chronic intra-erythrocytic infections in mammals and infectious endocarditis in dogs [39] and can also cause fever and myalgia in humans [40]. The diversity of *Bartonella* species in the fleas from our study is comparable with the diversity in fleas from rodents in Germany [36]. The relatively high prevalence of *Bartonella* spp. In fleas and rodents, which has been demonstrated in Slovakia, as well as in other European countries, supports the assumption that fleas are involved in maintaining the infection cycle in nature. Fleas are capable of serving as reservoirs and vectors for bacteria in different habitat types, and this is also supported by the results of Morick et al. [41], who showed that naturally infected rodents remained persistently infected with *Bartonella* for at least 89 days, and fleas could acquire *Bartonella* from wild rodents but could not transmit them transovarially.

The reservoir role of wild rodents in the rickettsiae life cycle has not been definitively confirmed, although it has been investigated across Europe. *Rickettsia* infections have been analyzed in the tissue and/or blood samples of rodents and in their ectoparasites. Rickettsiae in rodent ectoparasites could be acquired via blood meals from the infected hosts or by co-feeding from infected parasites. The prevalence of *Rickettsia* spp. was found in a range of 2–43% in different groups of ectoparasites [2,19,42,43,44,45,46,47,48]. The 19.1% rickettsial prevalence in fleas examined in the present study is in the above range, while only 2.5–10.8% of the tested fleas recovered from the rodents in eastern and southern Slovakia were *Rickettsia*-positive [19,20,48]. *Rickettsia helvetica* was the most frequently identified species in the fleas, which is in accordance with previous findings [2,44,47,48] and supports the hypothesis of the role of rodents as reservoir hosts for this species. The presence of *R. monacensis*, *R. felis*, and rickettsiae belonging to cluster of *R. slovaca*, *R. raoultii* and rickettsial ensymbionts were also identified in the ectoparasites in the above-mentioned studies, but detection of bacterial DNA in these fleas may be the result of an accidental uptake through feeding on infected hosts [44]. Rickettsiae were found mainly in *C. solutus*, *C. agyrtes*, and *N. fasciatus*, which are dominant parasites of the *Apodemus* species and *M. glareolus* in lower vegetation zones [49,50]. However, the vector role of fleas for the *Rickettsia* species mentioned above is still unclear and needs further investigation.

The role of rodents as a reservoir for *B. microti*, and particularly of the bank vole *M. glareolus* for *Hepatozoon* sp., has been confirmed in previous studies [51,52]. The infection of rodents and *I. ricinus* ticks with *B. microti* has been demonstrated using the same study sites as those included in the present study [23]. Although *B. microti* was found in fleas in Slovakia for the first time, detections of *B. microti* DNA in fleas changing hosts and feeding on rodents in natural foci of the parasite is not surprising. Yet this does not confirm either the vector or the reservoir role of fleas. On the other hand, fleas are known as definitive hosts for the sexual development of *Hepatozoon* spp., while rodents serve as intermediate hosts for the asexual development of the parasite. *Hepatozoon erhardovae* belongs to the most common apicomplexan blood and tissue parasites in the bank voles of Europe [26]. For example, in Hungary, an 11.45% infection rate was found in bank voles and 10.7% in fleas recovered from small rodents [27]. The same infection rate with *Hepatozoon* sp. was also confirmed in bank voles from the study sites in SW Slovakia [25]. The species spectrum of fleas infected with *Hepatozoon* sp. in Hungary and Slovakia was the same (*C. agyrtes*, *C. asimilis*, *M. turbidus*). Overall, our results confirmed previous findings on the role of fleas in the transmission cycle of *Hepatozoon* spp. infecting rodents.

## 4. Material and Methods

### 4.1. Origin of the Samples

Rodents and rodent-attached ectoparasites were collected from three study sites in Slovakia. The locations were described in detail in Kazimírová et al. [53] and Berthová et al. [11]. Briefly, Bratislava and Fúgelka are located in the Small Carpathian Mountains in SW Slovakia. The mountains are partially densely forested with sessile oak (*Quercus petraea*), with European hornbeam (*Carpinus betulus*) dominating at lower altitudes and European beech (*Fagus sylvatica*) dominating at higher altitudes. Bratislava, the recreational forest city park Železná studnička, and the campus of the Slovak Academy of Sciences (202–334 m above sea level, asl) represent a suburban habitat with significant anthropogenic impact. The natural habitat at Fúgelka (336–436 m asl) is set in a deciduous forest in the Small Carpathian Mountains. The Prievidza district is located in central Slovakia (289 m asl) and represents a forest-steppe rural area with Carpathian oak-hornbeam woods.

A total of 640 rodents from 6 species: yellow-necked mouse *Apodemus flavicollis* (Melchior, 1834), wood mouse *Apodemus sylvaticus* (L., 1758), bank vole *Myodes glareolus* (Schreber, 1780), common vole *Microtus arvalis* (Pallas, 1778), European pine vole *Microtus subterraneus* (de Selys-Longchamps, 1836), and Eurasian harvest mouse *Micromys minutus* (Pallas, 1771), were live-trapped during April–June and September–October, 2012–2014, in the suburban and natural habitats, and during May–July, and September–October, 2012–2013, in the rural habitat, using Sherman live traps baited with oat flakes (for details see [14,54]). Rodent trapping and handling were approved by the Ministry of Environment of the Slovak Republic, Regional Environmental Office in Bratislava (licence ZPO-594/2012-SAB). Each rodent individual was examined for the presence of ectoparasites. Collected ectoparasites were stored in 70% ethanol. Fleas were identified according to Rosický [55].

### 4.2. DNA Extraction and Molecular Analysis

From each parasitized rodent, a maximum of five fleas (of the same species) were selected for molecular analyses. In total, 665 flea specimens (388, 232, and 45 from the suburban, natural, and rural habitat, respectively) were analyzed. Fleas were washed with sterile water, dried, transferred to individual tubes, and crushed with a sterile carbon steel surgical scalpel blade (Surgeon, JAI Surgicals Lim., India). DNA from the fleas was extracted using Dneasy Blood and Tissue Kit (Qiagen) or NucleoSpin Tissue Kit (Macherey Nagel) following the manufacturer’s recommendations. The quantity and quality of DNA was assessed by NanoPhotometer Pearl (Implen, Germany). DNA samples were stored at −20 °C and later used as templates for the PCR amplifications. Flea samples were screened by PCR-based methods for the presence of *Bartonella* spp., *Rickettsia* spp., *Babesia* spp. and *Hepatozoon* spp. (for details see Supplementary File S2).

The presence of *Bartonella* spp. DNA was examined by a PCR assay targeting the 16S–23S rRNA gene intergenic spacer region (ITS), which generated a product of 420–780 bp [56]. Positive (*Bartonella* spp. DNAs from infected rodents confirmed by sequencing, obtained in a previous study [9]), and negative (nuclease-free water) controls were used in each PCR reaction.

Four sets of primers for amplifying the citrate synthase gene (*gltA*), 17-kDa antigen gene (17-kDa), outer membrane protein A gene (*ompA*), and PS120-protein encoding gene (gene D) were used to identify the species of *Rickettsia* [57,58,59,60]. DNA from *Rickettsia*-free ticks and nuclease-free water were used as negative controls in each reaction. DNAs from *R. helvetica* and *R. slovaca*, originating from ticks and previously sequenced, were used as positive controls. Additionally, the 23S rRNA gene was used to confirm the presence of *R. helvetica* in *Rickettsia*-positive flea samples using TaqMan PCR assay [61]. Each run of TaqMan PCR reactions included a negative template control, a positive control, and DNA standards containing 3 × 10^0^–3 × 10^6^ target copies with a sensitivity of 3 copies of the DNA.

The presence of *Babesia* spp. and *Hepatozoon* spp. DNA was detected by PCR amplification of a ~450 bp region of the 18S rRNA gene [62]. Positive and negative controls—*Babesia* spp. DNA from infected rodents confirmed by sequencing (obtained from [23]), and nuclease-free water, respectively, were used in each PCR run.

*Bartonella*-positive, *Rickettsia*-positive, and *Babesia*-positive amplicons were purified and analyzed via sequencing in both directions using Macrogen Inc. (Amsterdam, The Netherlands). The DNA sequences were compared with those available in GenBank using the Basic Local Alignment Search Tool (BLAST) (http://blast.ncbi.nlm.nih.gov; accessed 1 October 2018). New sequences generated in this study were submitted to the GenBank database under accession numbers MK239960-MK239961 and MH784529-MH784535 (Table S3). A phylogenetic tree was constructed using the Neighbor-Joining method [63] and a phylogenetic analysis was further performed using MEGA5 software [64].

### 4.3. Statistical Analysis

The prevalence and mean intensity of infestation (of rodents by fleas) were calculated according to Margolis et al. [65]. Differences in the prevalence of bacteria between male and female fleas, and between habitats were analyzed using Fisher’s exact test with an online calculator (http://www.socscistatistics.com; accessed 1 December 2018). A *p* Value < 0.05 was considered significant. Ninety-five percent confidence intervals (CI) were calculated using an online calculator (http://epitools.ausvet.com.au; accessed 1 December 2018).

## 5. Conclusions

The present study provides evidence of the presence of zoonotic pathogens in fleas parasitizing the wild-living small mammals of southwestern and central Slovakia and contributes to our knowledge of the ecology and genomic diversity of *Bartonella*, *Rickettsia*, *Babesia*, and *Hepatozoon*. *Bartonella* spp., identified in fleas, are the same as those detected previously in small mammals from the same study sites, suggesting that fleas are involved in the circulation of these bacteria in natural foci. On the other hand, our findings on the presence of tick-borne *Rickettsia* spp. and *Babesia microti* in fleas are not sufficient to prove the role of fleas in the transmission cycles. Still, the presence of zoonotic *Rickettsia* and *Babesia* species in fleas should be taken with caution. Ectoparasites from free-living and domestic animals should be screened continuously to detect new and emerging infectious agents and discover their role in the circulation of different vector-borne pathogens. Although no flea species is specifically associated with humans, fleas, together with ticks and other ectoparasites, live in close association with domestic animals and humans, who may come into contact with potentially infected fleas and acquire zoonotic infections.

## Figures and Tables

**Table 1:** Prevalence of *Bartonella* spp. in rodent-attached fleas from three habitats in Slovakia during 2012–2014 [no. of infected/ no. of collected (prevalence %)].

Site (Habitat)	Species	Male	Female	Total
Prievidza	*C. agyrtes*	1/2 (50)	2/5 (40)	3/7 (42.86)
(rural)	*C. assimilis*	3/3 (100)	5/5 (100)	8/8 (100)
	*C. solutus*	3/6 (50)	15/16 (93.75)	18/22 (81.82)
	*M. turbidus*		1/1 (100)	1/1 (100)
	*N. fasciatus*	6/7 (85.71)		6/7 (85.71)
	Subtotal	13/18 (72.22)	23/27 (85.19)	36/45 (80)
Fúgelka	*C. agyrtes*	42/101 (41.58)	41/93 (44.09)	83/194 (42.78)
(natural)	*C. bisoctodentatus*	1/1 (100)		1/1 (100)
	*C. congener*	8/13 (61.54)	6/16 (37.5)	14/29 (48.28)
	*C. sciurorum*		0/1 (0)	0/1 (0)
	*M. turbidus*	0/1 (0)	1/1 (100)	1/2 (50)
	*P. fallax*		0/1 (0)	0/1 (0)
	*P. similis*	1/1 (100)		1/1 (100)
	*R. integella*	0/1 (0)	1/2 (50)	1/3 (33.33)
	Subtotal	52/118 (44.07)	49/114 (42.98)	101/232 (43.53)
Bratislava	*C. agyrtes*	17/116 (14.66)	39/162 (24.07)	56/278 (20.14)
(suburban)	*C. congener*	6/13 (46.15)	8/26 (30.77)	14/39 (35.9)
	*C. sciurorum*	3/3 (100)	0/1 (0)	3/4 (75)
	*C. solutus*	6/22 (27.27)	9/34 (26.47)	15/56 (27.79)
	*H. talpae*	0/2 (0)	0/1 (0)	0/3 (0)
	*M. turbidus*		1/1 (100)	1/1 (100)
	*P. fallax*	0/3 (0)	0/4 (0)	0/7 (0)
	Subtotal	32/159 (20.13)	57/229 (24.89)	89/388 (22.94)
Total		97/295 (32.88)	129/370 (34.86)	226/665 (33.98)

**Table 2:** Prevalence of *Rickettsia* spp. in rodent-attached fleas from three habitats in Slovakia during 2012–2014 [no. of infected/ no. of collected (prevalence %)].

Site (Habitat)	Species	Male	Female	Total
Prievidza	*C. agyrtes*	1/2 (50)	0/5 (0)	1/7 (14.29)
(rural)	*C. assimilis*	1/3 (33.33)	2/5 (40)	3/8 (37.5)
	*C. solutus*	5/6 (83.3)	12/16 (75)	17/22 (77.27)
	*M. turbidus*		1/1 (100)	1/1 (100)
	*N. fasciatus*	4/7 (57.14)		4/7 (57.14)
	Subtotal	11/18 (61.11)	15/27 (55.56)	26/45 (57.78)
Fúgelka	*C. agyrtes*	1/101 (0.99)	1/93 (1.08)	2/194 (1.03)
(natural)	*C. bisoctodentatus*	0/1 (0)		0/1 (0)
	*C. congener*	0/13 (0)	0/16 (0)	0/29 (0)
	*C. sciurorum*		0/1 (0)	0/1 (0)
	*M. turbidus*	0/1 (0)	0/1 (0)	0/2 (0)
	*P. fallax*		0/1 (0)	0/1 (0)
	*P. similis*	0/1 (0)		0/1 (0)
	*R. integella*	0/1 (0)	0/2 (0)	0/3 (0)
	Subtotal	1/118 (0.85)	1/114 (0.88)	2/232 (0.86)
Bratislava	*C. agyrtes*	39/116 (33.62)	33/162 (20.37)	72/278 (25.9)
(suburban)	*C. congener*	2/13 (15.38)	7/26 (26.95)	9/39 (23.08)
	*C. sciurorum*	0/3 (0)	0/1 (0)	0/4 (0)
	*C. solutus*	6/22 (27.27)	12/34 (35.29)	18/56 (32.14)
	*H. talpae*	0/2 (0)	0/1 (0)	0/3 (0)
	*M. turbidus*		0/1 (0)	0/1 (0)
	*P. fallax*	0/3 (0)	0/4 (0)	0/7 (0)
	Subtotal	47/159 (29.56)	52/229 (22.71)	99/388 (25.52)
Total		59/295 (20.0)	68/370 (18.38)	127/665 (19.1)

**Table 3:** Prevalence of apicomplexan parasites (*Babesia microti*, *Hepatozoon* sp.) in rodent-attached fleas from three habitats in Slovakia during 2012–2014 [no. of infected/ no. of collected (prevalence %)].

Site (Habitat)	Species	Male	Female	Total
Prievidza	*C. agyrtes*	0/2 (0)	0/5 (0)	0/7 (0)
(rural)	*C. assimilis*	1/3 (33.33)	0/5 (0)	1/8 (12.5)
	*C. solutus*	0/6 (0)	0/16 (0)	0/22 (0)
	*M. turbidus*		0/1 (0)	0/1 (0)
	*N. fasciatus*	0/7 (0)		0/7 (0)
	Subtotal	1/18 (5.56)	0/27 (0)	1/45 (2.22)
Fúgelka	*C. agyrtes*	8/101 (7.92)	8/93 (8.60)	16/194 (8.25)
(natural)	*C. bisoctodentatus*	0/1 (0)		0/1 (0)
	*C. congener*	1/13 (7.69)	1/16 (6.25)	2/29 (6.90)
	*C. sciurorum*		0/1 (0)	0/1 (0)
	*M. turbidus*	0/1 (0)	1/1 (100)	1/2 (50)
	*P. fallax*		0/1 (0)	0/1 (0)
	*P. similis*	1/1 (100)		1/1 (100)
	*R. integella*	0/1 (0)	0/2 (0)	0/3 (0)
	Subtotal	10/118 (8.47)	10/114 (8.77)	20/232 (8.62)
Bratislava	*C. agyrtes*	2/116 (1.72)	5/162 (3.09)	7/278 (2.52)
(suburban)	*C. congener*	0/13 (0)	1/26 (3.85)	1/39 (2.56)
	*C. sciurorum*	0/3 (0)	0/1 (0)	0/4 (0)
	*C. solutus*	0/22 (0)	0/34 (0)	0/56 (0)
	*H. talpae*	0/2 (0)	0/1 (0)	0/3 (0)
	*M. turbidus*		0/1 (0)	0/1 (0)
	*P. fallax*	0/3 (0)	0/4 (0)	0/7 (0)
	Subtotal	2/159 (1.26)	6/229 (2.62)	8/388 (2.06)
Total		13/295 (4.41)	16/370 (4.32)	29/665 (4.36)

**Table 4:** Co-infections in rodent-attached fleas from the three sites in Slovakia during 2012–2014. [no. of co-infected/ no. of collected (prevalence %)].

Habitat	*Bartonella* sp., *Rickettsia* sp.	*Bartonella* sp., Rickettsial Endosymbionts	*Bartonella* sp., Apicomplexa	*Rickettsia* sp., Apicomplexa	*Bartonella* sp., *Rickettsia* sp., Apicomplexa
Suburban	1	24	3	4	1
Natural	0	0	0	1	0
Rural	12	3	1	0	0
Total	13/665 (1.95)	27/665 (4.06)	4/665 (0.60)	5/665 (0.75)	1/665 (0.15)

## Data Availability

The datasets used and/or analyzed during the current study are available from the first and corresponding author upon request. New partial DNA sequences were submitted to the GenBank database under the accession numbers MK239960, MK239961, MH784529-MH784535.

## Supplementary Materials

The following supporting information can be downloaded at: https://www.mdpi.com/article/10.3390/pathogens11080886/s1, Supplementary File S1: Estimates of evolutionary divergence between sequences in the *Bartonella taylorii* cluster. Standard error estimate(s) are shown above the diagonal and were obtained by a bootstrap procedure (1000 replicates). Analyses were conducted using the maximum composite likelihood model [66]; Supplementary File S2: PCR protocols for detection and identification of *Bartonella*, *Rickettsia* and *Babesia* species; Table S1: Numbers of captured rodents, prevalence and mean intensity of their infestation with fleas in three habitat types in southwestern and central Slovakia; Table S2: List of flea species collected from individual rodent species in three habitat types in southwestern and central Slovakia; Table S3: GenBank accession numbers of *Bartonella* spp. 16S–23S rRNA ITS, *Babesia microti* and *Hepatozoon* sp. 18S rRNA gene sequences identified in fleas collected from rodents; Figure S1. Phylogenetic tree inferred from comparison of the *Bartonella* 16S–23S internal transcribed spacer sequences. The evolutionary history was inferred using the neighbour-joining method. The percentages of replicate trees in which the associated taxa were clustered together via the bootstrap test (1000 replicates) are shown next to the branches [67]. GenBank accession numbers are included. BA2-BA219—fleas were collected from rodents in Bratislava, FU2-FU180—fleas were collected from rodents in Fúgelka, PD5-PD33—fleas were collected from rodents in Prievidza. Sequences indicated by black dots were submitted to GenBank; Figure S2. Phylogenetic tree inferred from comparison of the *Rickettsia* gltA sequences. The evolutionary history was inferred using the neighbor-joining method. The percentage of replicate trees in which the associated taxa were clustered together via the bootstrap test (1000 replicates) are shown next to the branches [67]. GenBank accession numbers are included. Samples marked as BA—fleas were collected from rodents in Bratislava, FU—fleas were collected from rodents in Fúgelka, PD—fleas were collected from rodents in Prievidza; Figure S3. Phylogenetic tree inferred from comparison of the *Rickettsia* 17-kDA sequences. The evolutionary history was inferred using the neighbor-joining method. The percentage of replicate trees in which the associated taxa were clustered together via the bootstrap test (1000 replicates) are shown next to the branches [67]. GenBank accession numbers are included. Samples marked as BA—fleas were collected from rodents in Bratislava, FU—fleas were collected from rodents in Fúgelka, PD—fleas were collected from rodents in Prievidza.
