# Supplementary material for: Bartonella, Rickettsia, Babesia, and Hepatozoon Species in Fleas (Siphonaptera) Infesting Small Mammals of Slovakia (Central Europe)
Source: Pathogens. 2022 Aug 6;11(8):886. doi: 10.3390/pathogens11080886 (PMC9413308; doi:10.3390/pathogens11080886)
Supplement: Supplementary file 1 [file pathogens-11-00886-s001.zip › Supplementary file S2.pdf]

Supplementary file S2. PCR protocols for detection and identification of *Bartonella, Rickettsia, Babesia* and *Hepatozoon* species

PCR protocol for the detection of *Bartonella* spp. based on 16S–23S rRNA gene intergenic spacer region [1]

Amplification was performed in a thermocycler Labcycler, SensoQuest, Germany.

PCR reactions were carried out in a volume of 20 µl containing 2 µl of DNA template and 10 µl of Master mix (SuperHot PCR Master Mix, Bioron, Germany), 0.2 µl of each primer (10 µM), 0.5 µl of 100 mM $MgCl_2$ and 7.1 µl of nuclease free water. The thermal cycle reaction consisted of an initial 2 min denaturation at 95°C, followed by 40 cycles at 94°C for 30 s, 66°C for 30 s, and 72°C for 30 s, with a final extension at 72°C for 5 min.

PCR protocol for the detection of *Rickettsia* spp.

The PCR equipment was a Labcycler, SensoQuest, Germany.

1. PCR amplification to detect *Rickettsia* spp. based on the *gltA* gene [2]

Identification of *Rickettsia* spp. was performed by the nested PCR amplification of the genus specific *gltA* gene using the oligonucleotide pairs RpCS.877p and RpCS.1258n for the primary PCR amplification and RpCS.896p and RpCS.1233n for the secondary amplification. The primary amplification of the specimen was performed in final reaction volume of 20 µl containing 2 µl of DNA template and 10 µl of Master mix (SuperHot PCR Master Mix, Bioron, Germany), 0.2 µl of each primer (10 pmol), 0.5 µl of 100 mM $MgCl_2$ and 7.1 µl of nuclease free water, and PCR cycling condition consisted of incubation at 95°C for 5 min, then 35 cycles each of 15 s at 95°C, 15 s at 54°C, and 30 s at 72°C, followed by a final extension cycle of 3 min at 72°C. After this, 1 µl of the amplified product was again amplified in the nested PCR with inner primer set and the same cycling condition.

2. PCR amplification to detect *Rickettsia* spp. based on the *17-kDa* gene [3]

Eeach reaction consisted of 2 µl of genomic DNA, 10 µl of Master mix (SuperHot PCR Master Mix, Bioron, Germany) 0.5 µl of each primer (10 pmol) (17-kDa-5, 17-kDa-3), 0.5 µl of 100 mM $MgCl_2$ and 6.5 µl of nuclease free water. The cycling conditions consisted of an initial 2 min denaturation at 95°C, followed by 33 cycles at 95°C for 30 s, 61°C for 30 s, and 72°C for 30 s, with a final extension at 72°C for 8 min. After the first amplification, 1µl of the product

was reamplified, now using inner primers 17-kDa-1 and 17-kDa-2. The amplification program was the same with above.

3. PCR amplification to detect *Rickettsia* spp. based on the *ompA* gene [4]

The reaction mixture of a 20 µl reaction volume contained 2 µl of genomic DNA, 10 µl of Master mix (SuperHot PCR Master Mix, Bioron, Germany) 0.5 µl of each primer (10 pmol) (190-70F, 190-701R), 0.5 µl of 100 mM $MgCl_2$ and 6.5 µl of nuclease free water. The cycling conditions consisted of an initial 5 min denaturation at 95°C, followed by 35 cycles at 95°C for 30 s, 55°C for 45 s, and 72°C for 45 s, with a final extension at 72°C for 8 min.

4. PCR amplification to detect *Rickettsia* spp. based on the *sca4* gene [5]

The reaction mixture of a 20 µl reaction volume contained 2 µl of genomic DNA, 10 µl of Master mix (SuperHot PCR Master Mix, Bioron, Germany) 0.5 µl of each primer (10 pmol) (D767f, D1390r), 0.5 µl of 100 mM $MgCl_2$ and 6.5 µl of nuclease free water. The cycling conditions as followed: an initial 2 min denaturation at 95°C, followed by 35 cycles at 95°C for 20 s, 50°C for 30 s, and 72°C for 30 s, with a final extension at 72°C for 10 min.

5. TaqMan real-time PCR amplification to detect *Rickettsia helvetica* based on the 23S rRNA gene [6]

The real-time PCR equipment was a Bio-Rad CFX96™ Real-Time System.

As master mix, 10 µl of 2x Master Mix (Maxima Probe qPCR Master Mix, Thermo Scientific, USA) was mixed with 0.25 µl of TaqMan probe and 0.5 µl of both primers, 3 µl of DNA was added into each sample. The PCR program consisted of initial denaturation at 95 °C for 15 minutes, followed by 40 cycles of denaturation at 95°C for 15 seconds and annelation at 60 °C for 1 minute.

6. PCR protocol for the detection of *Babesia* spp. and *Hepatozoon* spp. based on amplification of a region of the 18S ribosomal RNA gene [7]

The PCR equipment was a BioRad T100™ Thermal Cycler, USA.

The PCR reaction was carried out in a final volume of 25 µl containing 5 µl of DNA template and 20 µl of PCR mix: 0.125 µl of HotStarTaq Plus DNA Polymerase (5 U/µl; Qiagen, Hilden, Germany), 0.5 µl of each primer (10 µM), 0.5 µl of dNTP (10 mM), 2.5 µl of Coral Load PCR buffer (containing 15 mM $MgCl_2$), 1 µl of $MgCl_2$ (25 mM) and 14.875 µl of nuclease free water. PCR cycling conditions were: an initial denaturation step at 95 °C for 5 min, 35 cycles each of

1 min at 94 °C, 1 min at 55 °C, and 2 min at 72 °C, followed by a final amplification step of 5 min at 72 °C. PCR products were separated by electrophoresis in a 1.5% agarose gel and treated with GoodView^{TM} Nucleic Acid stain (SBS Genetech, China) and visualized by UV transillumination. PCR positive samples were purified and analysed by sequencing with forward and reverse primers used for PCR amplification by Macrogen (Amsterdam, Netherland).

Nucleotide sequences of the primers used for the identification *Bartonella, Rickettsia, Babesia* and *Hepatozoon* spp. and lengths of the target fragments

Gene	Primer	Sequence(5’-3’)	Target fragment length (bp)	Reference
	Bartonella species			
16S–23S rRNA ITS	BA325s	CTTCAGATGATGATCCCAAGCCTTCTGGCG	420-780	1
	BA1100as	GAACCGACGACCCCCTGCTTGCAAAGCA		
	Rickettsia species			
gltA	RpCS.877p	GGGGGCCTGCTCACGGCGG	381	2
	RpCS.1258n	ATTGCAAAAAGTACAGTGAACA		
	RpCS.896p	GGCTAATGAAGCAGTGATAA		
	RpCS.1233n	GCGACGGTATACCCATAGC		
17-kDa	17K-5	GCTTTACAAAATTCTAAAAACCATATA	434	3
	17K-3	TGTCTATCAATTCACAACTTGCC		
	17KD1	GCTCTTGCAACTTCTAT GTT		
	17KD2	CATTGTTCGTCAGGTTGGCG		
ompA	190-70F	ATGGCGAATATTTCTCCAAAA	632	4
	190-701R	GTTCCGTTAATGGCAGCATCT		
sca4	D767f	CGATGGTAGCATTAAAAGCT	590-653	5
	D1390r	CTTGCTTTTCAGCAATATCAC		
23S rRNA	Rh.147f	TTTGAAGGAGACACGGAACACA	65	6
	Rh. 211r	TCCGGTACTCAAATCCTCACGTA		
	Rh.170p	AACCGTAGCGTACACTTA-TAMRA		
	Babesia and *Hepatozoon* species			
	BJ1	GTCTTGTAATTGGAATGATGG	450	7
	BN2	TAGTTTATGGTTAGGACTACG		

References

1. Maggi, R.G.; Kosoy, M.; Mintzer, M.; Breitschwerdt, E.B. Isolation of *Candidatus* Bartonella melophagi from human blood. *Emerg. Infect. Dis.* **2009**, 15, 66–68.
2. Choi, Y.J.; Jang, W.J.; Kim, J.H.; Ryu, J.S.; Lee, S.H.; Park, K.H.; Paik, H.S.; Koh, Y.S.; Chi, M.S.; Kim, I.S. Spotted fever group and typhus group rickettsioses in humans, South Korea. *Emerg. Infect. Dis.* **2005**, 11, 237-244.
3. Anstead, C.A.; Chilton, N.B. A novel *Rickettsia* species detected in Vole Ticks (*Ixodes angustus*) from Western Canada. *Appl. Environ. Microbiol.* **2013,** 79, 7583-7589.
4. Zhang, L.; Jin, J.; Fu, X.; Raoult, D.; Fournier, P.E. Genetic differentiation of Chinese isolates of *Rickettsia sibirica* by partial ompA gene sequencing and multispacer typing. *J.*

Clin. Microbiol. **2006,** 44, 2465-2467.

5. Sekeyová, Z; Roux, V.; Raoult, D. Phylogeny of *Rickettsia* spp. inferred by comparing sequences of ‘gene D’, which encodes an intracytoplasmic protein. *Int. J. Syst. Evol. Microbiol.* **2001**, 51, 1353–1360.
6. Boretti, F.S.; Perreten, A.; Meli, M.L.; Cattori, V.; Willi, B.; Wengi, N.; Hornok, S.; Honegger, H.; Hegglin, D.; Woelfel, R.; Reusch, C.E.; Lutz, H.; Hofmann-Lehmann, R. Molecular investigations of *Rickettsia helvetica* infection in dogs, foxes, humans, and *Ixodes* ticks. *Appl. Environ. Microbiol.* **2009,** 75, 3230-3237.
7. Casati, S.; Sager, H.; Gern, L.; Piffaretti, J.C. Presence of potentially pathogenic *Babesia* sp. for human in *Ixodes ricinus* in Switzerland. *Ann. Agric. Environ. Med.* **2006,** 13, 65–70.
