# Supplementary material for: Bartonella, Rickettsia, Babesia, and Hepatozoon Species in Fleas (Siphonaptera) Infesting Small Mammals of Slovakia (Central Europe)
Source: Pathogens. 2022 Aug 6;11(8):886. doi: 10.3390/pathogens11080886 (PMC9413308; doi:10.3390/pathogens11080886)
Supplement: Supplementary file 1 [file pathogens-11-00886-s001.zip › Table S1.pdf]

Table S1. Numbers of captured rodents (N), numbers of infested rodents (NP), numbers of fleas (n), and prevalence (P%) and mean intensity (I) of rodents´ infestation with fleas in three habitat types in southwestern and central Slovakia

Habitat type, site	Rodent species	N	NP	n	P (%)	I
Suburban Bratislava						
2012	*A. flavicollis*	90	37	56	41.11	1.51
	A. sylvaticus	1	0	0	-	-
	M. glareolus	94	34	56	36.17	1.65
2013	*A. flavicollis*	5	4	15	80.00	3.75
	M. glareolus	1	0	0	-	-
2014	*A. flavicollis*	85	61	195	71.76	3.20
	M. glareolus	24	21	112	87.50	5.33
Total		300	157	434	52.33	2.76
Natural Fúgelka						
2012	*A. flavicollis*	117	48	88	41.02	1.83
	M. glareolus	84	36	98	42.86	2.72
	M. arvalis	19	5	16	26.37	3.2
	M. subterraneus	1	1	3	100	3
2013	*A. flavicollis*	1	0	0	-	-
	M. glareolus	1	0	0	-	-
2014	*A. flavicollis*	58	20	31	34.48	1.55
	M. glareolus	23	11	21	47.83	1.91
	M. minutus	1	0	0	-	-
Total		305	121	257	39.67	2.12
Rural Prievidza						
2012	*A. flavicollis*	40	17	29	42.50	1.71
	M. glareolus	12	3	4	25.00	1.33
2013	*A. flavicollis*	5	3	10	60.00	3.33
Total		57	23	43	40.35	1.87
TOTAL (all sites)		662	301	734	45.47	2.44
