# Supplementary material for: Bartonella, Rickettsia, Babesia, and Hepatozoon Species in Fleas (Siphonaptera) Infesting Small Mammals of Slovakia (Central Europe)
Source: Pathogens. 2022 Aug 6;11(8):886. doi: 10.3390/pathogens11080886 (PMC9413308; doi:10.3390/pathogens11080886)
Supplement: Supplementary file 1 [file pathogens-11-00886-s001.zip › Table S2.pdf]

**Table S2**. List of flea species collected from individual rodent species in three habitat types in southwestern and central Slovakia

| Habitat type, site | Rodent species | Flea species |
|---|---|---|
| Suburban, Bratislava | | |
| | *Apodemus flavicollis* | *Ctenophthalmus agyrtes* |
| | | *Ctenophthalmus congener* |
| | | *Ctenophthalmus solutus* |
| | | *Ceratophylus sciurorum* |
| | | *Hystrichopsylla talpae* |
| | *Myodes glareolus* | *Ctenophthalmus agyrtes* |
| | | *Ctenophthalmus congener* |
| | | *Ctenophthalmus solutus* |
| | | *Ceratophylus sciurorum* |
| | | *Peromyscopsylla fallax* |
| | | *Megabothris turbidus* |
| | | *Hystrichopsylla talpae* |
| Natural, Fúgelka | | |
| | *Apodemus flavicollis* | *Ctenophthalmus agyrtes* |
| | | *Ctenophthalmus congener* |
| | | *Ceratophylus sciurorum* |
| | | *Palaeopsylla similis* |
| | *Myodes glareolus* | *Ctenophthalmus agyrtes* |
| | | *Ctenophthalmus congener* |
| | | *Ctenophthalmus bisoctodentatus* |
| | | *Megabothris turbidus* |
| | | *Rhadinopsylla integella* |
| | | *Peromyscopsylla fallax* |
| | *Microtus arvalis* | *Ctenophthalmus agyrtes* |
| | | *Ctenophthalmus congener* |
| | *Microtus subterraneus* | *Ctenophthalmus agyrtes* |
| Rural, Prievidza | | |
| | *Apodemus flavicollis* | *Ctenophthalmus agyrtes* |
| | | *Ctenophthalmus solutus* |
| | | *Ctenophthalmus assimilis* |
| | | *Nosopsyllus fasciatus* |
| | | *Megabothris turbidus* |
| | *Myodes glareolus* | *Ctenophthalmus agyrtes* |
| | | *Ctenophthalmus solutus* |
| | | *Ctenophthalmus assimilis* |
