# Supplementary material for: Bartonella, Rickettsia, Babesia, and Hepatozoon Species in Fleas (Siphonaptera) Infesting Small Mammals of Slovakia (Central Europe)
Source: Pathogens. 2022 Aug 6;11(8):886. doi: 10.3390/pathogens11080886 (PMC9413308; doi:10.3390/pathogens11080886)
Supplement: Supplementary file 1 [file pathogens-11-00886-s001.zip › Table S3.pdf]

**Table S3.** GenBank accession numbers of *Bartonella* spp. 16S–23S rRNA ITS, *Babesia microti* and *Hepatozoon* sp. 18S rRNA gene sequences identified in fleas collected from rodents

| Name of the isolate | Source | bp | GenBank accession number | |
|---|---|---|---|---|
| | | | 16S–23S rRNA ITS | 18S rRNA |
| Uncultured *Bartonella* sp. clone FU184 | *Ctenophthalmus agyrtes* from *Myodes glareolus* | 460 | MK239960 | |
| Uncultured *Bartonella* sp. clone BA37 | *Ctenophthalmus solutus* from *Apodemus flavicollis* | 497 | MK239961 | |
| *Hepatozoon* sp. SK1 isolate F73 | *Ctenophthalmus agyrtes* from *Myodes glareolus* | 502 | | MH784529 |
| *Hepatozoon* sp. SK1 isolate F103 | *Ctenophthalmus agyrtes* from *Apodemus flavicollis* | 502 | | MH784530 |
| *Hepatozoon* sp. SK1 isolate F114 | *Ctenophthalmus agyrtes* from *Apodemus flavicollis* | 502 | | MH784531 |
| *Hepatozoon* sp. SK1 isolate F185 | *Ctenophthalmus agyrtes from Myodes glareolus* | 502 | | MH784532 |
| *Babesia microti* isolate F85 | *Ctenophthalmus agyrtes* from *Myodes glareolus* | 474 | | MH784533 |
| *Babesia microti* isolate F92 | *Ctenophthalmus congener* from *Myodes glareolus* | 474 | | MH784534 |
| *Babesia microti* isolate F110 | *Palaeopsylla similis* from *Apodemus flavicollis* | 474 | | MH784535 |
