# Supplementary figures and images for: Bartonella, Rickettsia, Babesia, and Hepatozoon Species in Fleas (Siphonaptera) Infesting Small Mammals of Slovakia (Central Europe)
Source: Pathogens. 2022 Aug 6;11(8):886. doi: 10.3390/pathogens11080886 (PMC9413308; doi:10.3390/pathogens11080886)

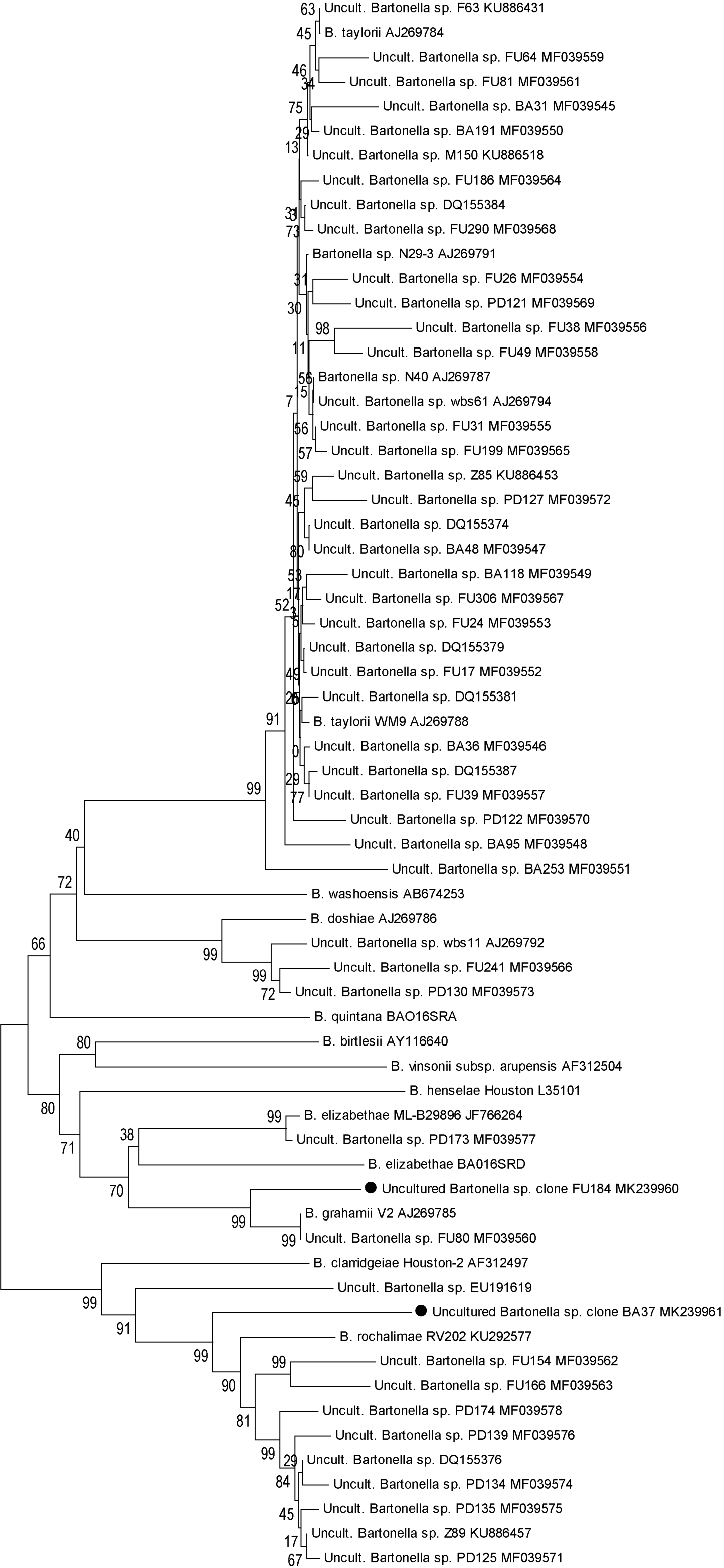

0.05

Supplement: Supplementary file 1 [file pathogens-11-00886-s001.zip › Figure. S1.pdf]

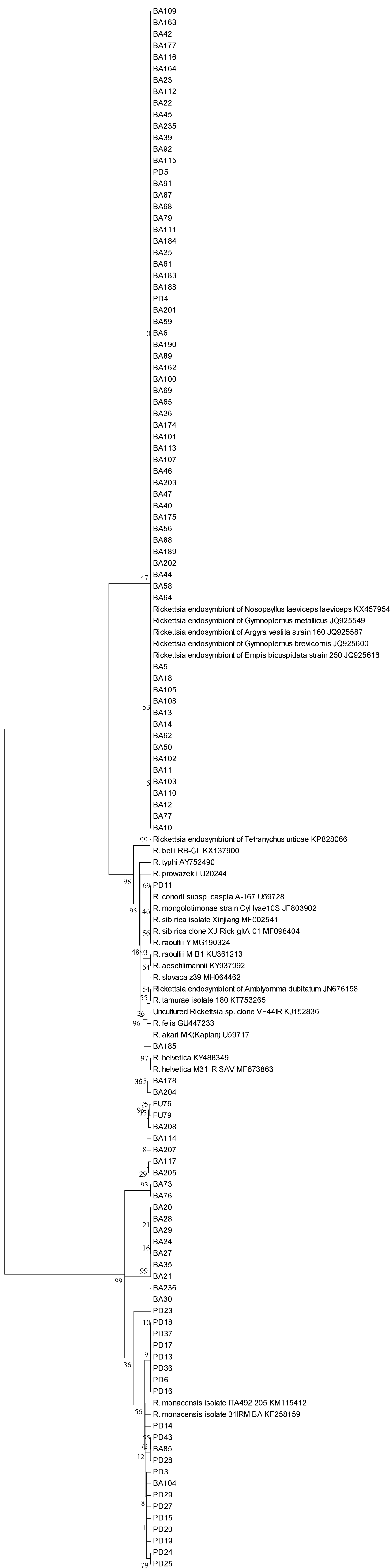

Supplement: Supplementary file 1 [file pathogens-11-00886-s001.zip › Figure. S2.pdf]

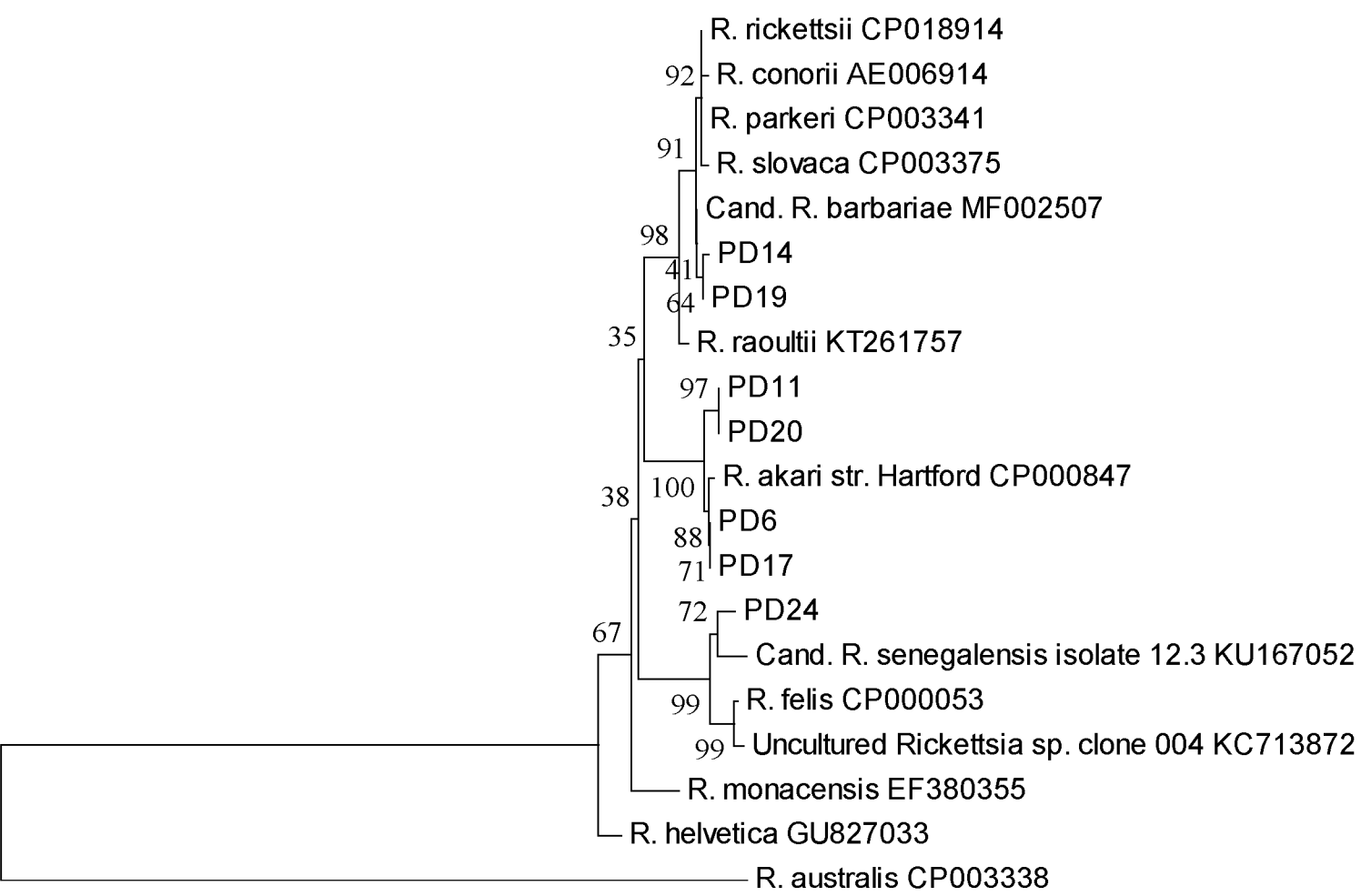

0.05

Supplement: Supplementary file 1 [file pathogens-11-00886-s001.zip › Figure. S3.pdf]
